# Hydroxyacid Oxidase 1, a Glutamine Metabolism-Associated Protein, Predicts Poor Patient Outcome in Luminal Breast Cancer

**DOI:** 10.3390/ijms252111572

**Published:** 2024-10-28

**Authors:** Busra Erkan, Skye MacIntyre, Charlotte Brown, Ali Fakroun, Ayat G. Lashen, Nigel P. Mongan, Ian O. Ellis, Emad A. Rakha, Andrew R. Green

**Affiliations:** 1 Nottingham Breast Cancer Research Centre, Academic Unit for Translational Medical Sciences, School of Medicine, University of Nottingham Biodiscovery Institute, University Park, Nottingham NG7 2RD, UKemad.rakha@nottingham.ac.uk (E.A.R.); 2 School of Veterinary Medicine and Science, University of Nottingham Biodiscovery Institute, University Park, Nottingham NG7 2RD, UK; 3 Cellular Pathology, Nottingham University Hospitals NHS Trust, Nottingham City Hospital, Hucknall Road, Nottingham NG5 1PB, UK

**Keywords:** luminal breast cancer, oestrogen receptor, hydroxyacid oxidase 1, solute carriers, prognostic significance

## Abstract

Breast cancer (BC), which remains the most prevalent malignancy among women, is characterised by significant heterogeneity across its molecular subtypes. Oestrogen receptor-positive (ER+) (luminal) BC represents approximately 75% of cases, and despite advancements in treatment there remains around a 40% recurrence rate. Cellular uptake of glutamine is conducted by solute carriers (SLCs), which are significantly associated with outcome in luminal BC. In this study, differential gene expression analysis was carried out using The Cancer Genome Atlas BC dataset. This identified hydroxyacid oxidase 1 (*HAO1*) as significantly overexpressed in luminal BC with a high expression of SLCs. Extended analysis in the METABRIC (n = 1980) and Breast Cancer Gene-Expression Miner (n = 4421) transcriptomic databases and the Nottingham (n = 952) BC tissue cohort showed a varied survival outcome for HAO1 expression at the genomic, transcriptomic, and proteomic levels. *HAO1* copy number (CN) gain (*p* = 0.002) and high HAO1 protein expression (*p* = 0.019) were associated with poor prognosis in luminal BC, whereas high *HAO1* mRNA expression correlated with better survival outcomes (*p* = 0.023) suggesting a complex regulatory mechanism affecting HAO1 at different biological levels. Importantly, in luminal BC patients treated with endocrine therapy, high protein expression of HAO1 predicted shorter distant-metastasis free survival (*p* = 0.042). The knockdown of *SLC1A5* and *SLC7A5* significantly reduced HAO1 expression in MCF-7 and ZR-751 BC cell lines. Protein analysis confirmed significant associations between HAO1 and SLC7A5 and SLC1A5, emphasising a potential role for the enzyme in glutamine metabolism and its potential as a therapeutic target. This study underscores the prognostic significance of HAO1 in luminal BC and its relationship with patient outcomes.

## 1. Introduction

Breast cancer (BC) is the most common malignancy and is a heterogeneous disease featuring several subtypes distinguished by the differences in morphology, response to therapy, and clinical outcomes [1]. The most common biological subtype, accounting for around 75% of all cases, is luminal or oestrogen receptor-positive (ER+) BC [2,3]. Despite advancements in treatments including endocrine therapy, there remains around a 40% recurrence rate [1]. The high heterogeneity of BC, therefore, calls for further insight into the molecular mechanisms and major drivers affecting luminal BC so that better patient outcomes are achieved with more accurate individualised therapy.

The particular emphasis that Warburg left in his 1956 study was that, despite the presence of oxygen, energy production by cancer cells occurs prevalently through glycolysis [4]. In 2014, Alberghina and Gaglio further advanced this understanding to describe the redox control of glutamine utilisation in cancer, with a highlighted critical role for glutamine in serving growth and survival signalling [5]. In this respect, the metabolic reprogramming of many tumour cells makes them highly dependent on glutamine; while some tumour cells can adapt to glutamine deprivation, many exhibit impaired growth and survival without an adequate glutamine supply, highlighting its importance in cancer metabolism.

Cellular uptake of glutamine is conducted by solute carriers (SLCs), and upon cell entry it is metabolised to glutamate by glutaminase [6]. In our prior work, we identified the co-expression of three predominant SLCs important in glutamine metabolism—namely, SLC1A5, SLC7A5, and SLC3A2—and that these are significantly overexpressed in aggressive luminal BC subgroups and are associated with poor outcome [7]. Although SLCs have significant potential as therapeutic targets, their characterisation remains a research focus. To create the potential effective targeted treatments, however, some challenges need to be addressed. The expression of SLCs is diverse, with only a limited number of SLCs showing restricted expression to specific cell types [8]. Furthermore, SLCs exhibit interchangeable properties regarding their substrate specificity; multiple substrates can be transported by more than one SLC, indicating that various SLCs might be responsible for the transport of a given substrate [9,10]. These factors complicate the specific pharmacological interventions, making targeted therapy more challenging. A comprehensive understanding of the signalling pathways involved in SLCs can be achieved through identification, which may subsequently lead to the discovery of additional therapeutic targets.

This study aims to assess the clinical relevance of HAO1 and its potential interactions with SLCs in the context of luminal BC.

## 2. Results

### 2.1. HAO1 Expression in Invasive Luminal Breast Cancer

Many tumours exhibited a normal *HAO1* gene CN, with only 165/1980 cases (8.3%) showing an *HAO1* gain and 28/1980 cases (1.4%) displaying a CN loss. There was no association between the *HAO1* CN and *HAO1* mRNA expression (*p* = 0.28).

**Figure 1:**
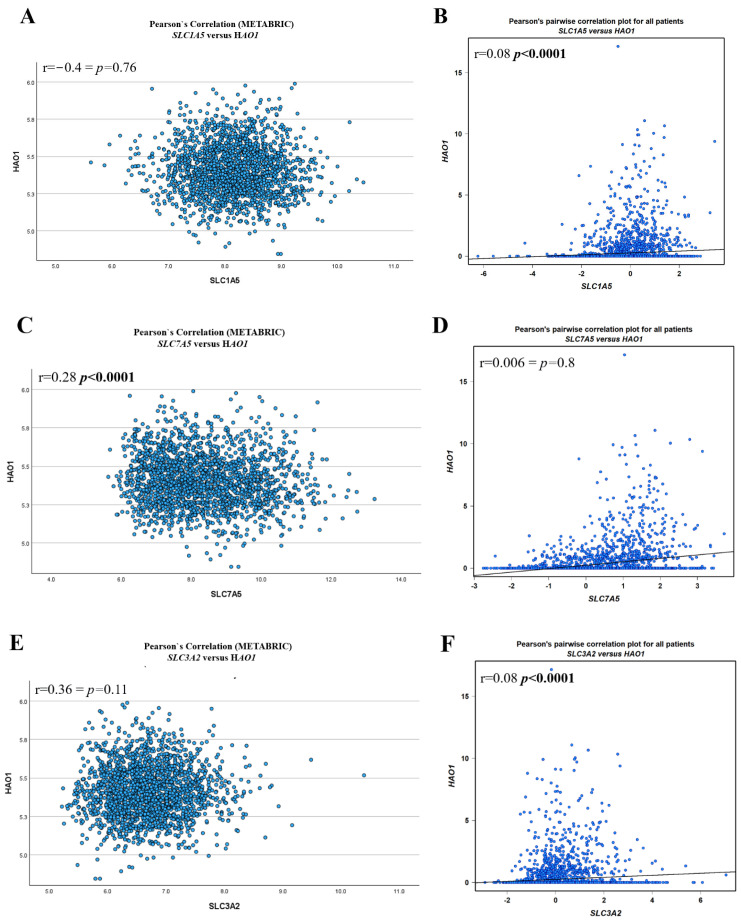
*HAO1* mRNA and SLC mRNA correlation using METABRIC (**A**,**C**,**E**) and bc-GenExMiner (**B**,**D**,**F**): (**A**,**B**) *SLC1A5*, (**C**,**D**) *SLC7A5*, (**E**,**F**) *SLC3A2.* Statistically significant *p*-values shown in **bold**.

A weak positive correlation was observed between *HAO1* mRNA and *SLC7A5* mRNA in the bc-GenExMiner dataset (r = 0.28, *p* < 0.0001) (Figure 1C), and a very weak positive association between *HAO1* mRNA and *SLC1A5* mRNA (r = 0.08, *p* < 0.0001) and *SLC3A2* mRNA (r = 0.08, *p* < 0.0001) (Figure 1B,F). There was a weak association between *HAO1* mRNA and *SLC7A5* mRNA in the METABRIC dataset (Figure 1C). Methylation of *HAO1* was seen at four CpG sites across the body, 3′UTR, TSS200, and 1st exon regions (Supplementary Figure S1A). In breast tumours, promoter methylation was significantly lower than that in normal breast tissue (Supplementary Figure S1B; *p* < 0.0001). There was also low methylation of cg19201723 in ER positive (luminal) compared with ER negative tumours (Supplementary Figure S1D) but not with the other CpG sites (Supplementary Figure S1C,E,F).

**Figure 2:**
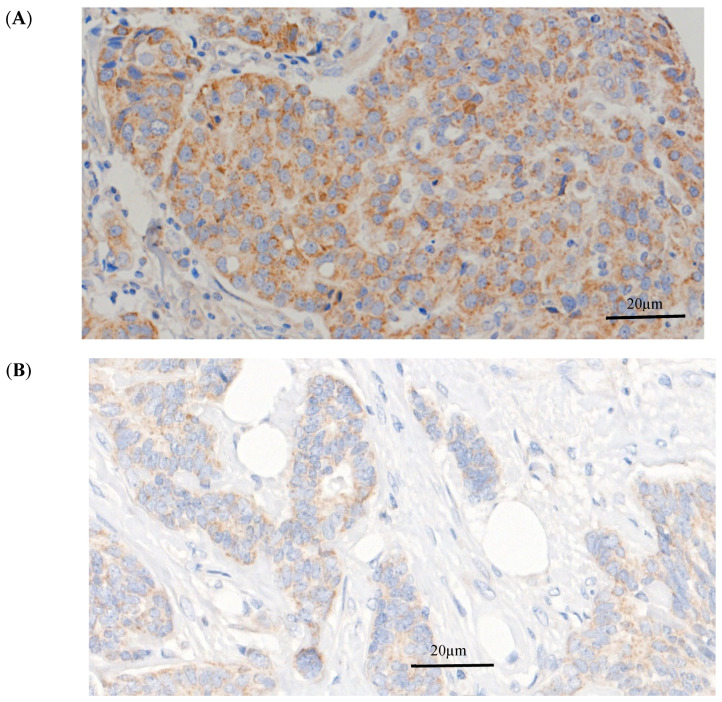
HAO1 protein expression in invasive breast cancer. (**A**) High HAO1 expression, (**B**) Negative HAO1 expression. Magnification 40×. Scale bars = 20 μm.

HAO1 protein expression was predominantly observed in the cytoplasm of invasive breast tumour cells, with expression levels ranging from absent to high (Figure 2). HAO1 immunoreactivity in full-face breast tissue sections showed homogenous staining of invasive BC cells, indicating that TMA cores were representative of the whole tumour. High HAO1 protein expression was observed in 378/1793 cases (21%). The distribution of HAO1 protein expression (H-scores) is shown in the histogram in Supplementary Figure S2.

HAO1 protein showed a positive association with SLC1A5 and SLC7A5 (Table 1; *p* = 0.0001 and *p* = 0.04, respectively) but not SLC3A2. Due to the different cohorts, it was not possible to correlate HAO1 protein expression with either *HAO1* CN or mRNA.

### 2.2. Impact of SLC Knockdown on HAO1 Expression

**Figure 3:**
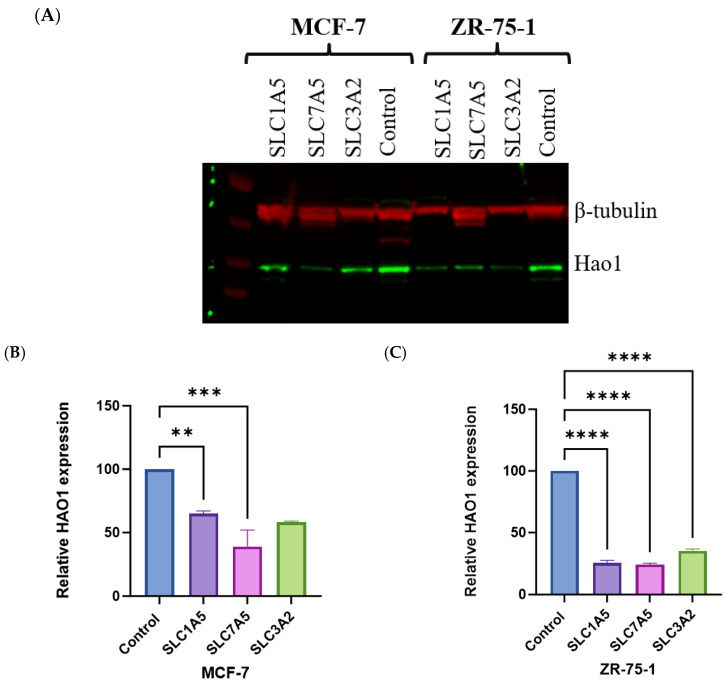
(**A**) Effect of SLC1A5, SLC7A5, and SLC3A2 knockdown on HAO1 expression in (**B**) MCF-7 and (**C**) ZR-75-1. β-tubulin was used as a loading control. Results shown are mean ± SE of two independent experiments. **** *p* < 0.0001; *** *p* < 0.001; ** *p* < 0.01.

HAO1 protein levels were reduced in both MCF-7 and ZR-75-1 cells in knockdown *SLC1A5* or *SLC7A5* compared to the control (Figure 3A). In MCF-7 cells, knockdown of *SLC1A5* resulted in a marked decrease in HAO1 expression, showing approximately a 40% reduction compared to the control (*p* < 0.01). *SLC1A5* knockdown led to an even more pronounced reduction in HAO1 levels, with expression decreasing to nearly 70% less than the control (*p* < 0.0001; Figure 3B,C). Similarly, knockdown of *SLC7A5* resulted in substantial decreases in HAO1 expression, with levels reduced to about 60% in MCF-7 and 70% in ZR-75-1 of the control (*p* < 0.001 and *p* < 0.0001, respectively; Figure 3B,C). Knockdown of *SLC3A2* showed a moderate 65% decrease in HAO1 expression in ZR-75-1 (*p* < 0.0001; Figure 3C).

### 2.3. Bioinformatics Analysis of HAO1 with Clinicopathological Invasive Breast Cancer Characteristics

**Figure 4:**
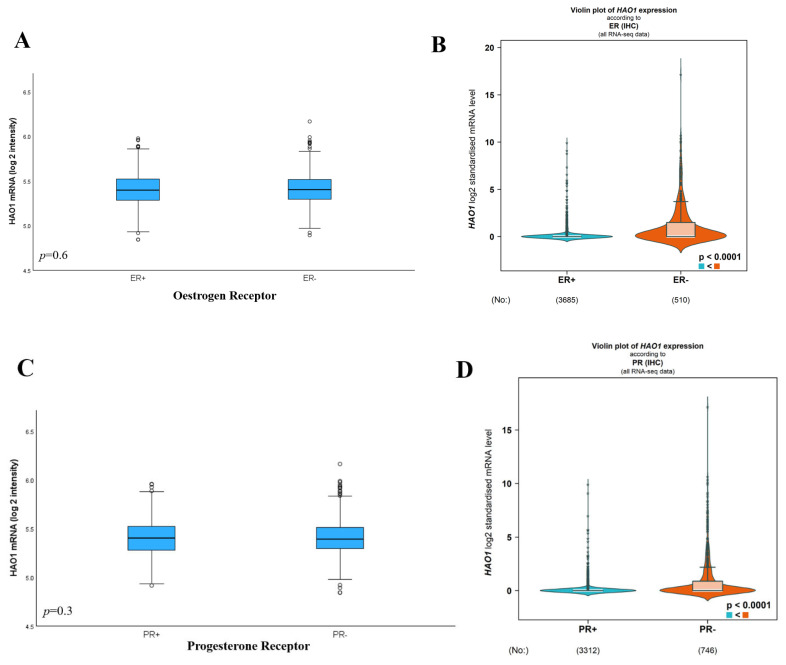

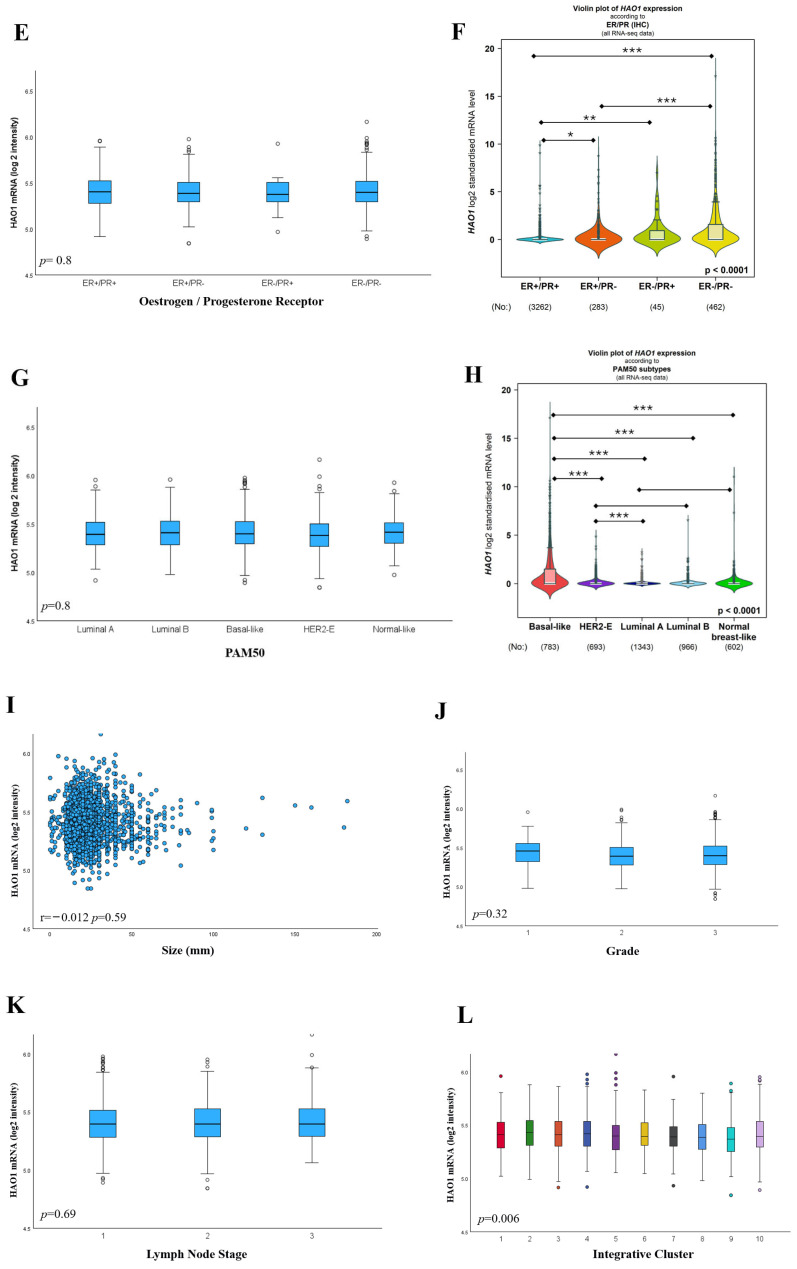
*HAO1* mRNA expression and its association with clinicopathological parameters and molecular subtypes in the METABRIC cohort was analysed using the one-way analysis of variance (ANOVA) with the post hoc Tukey test and using bc-GenExMiner, according to (**A**,**B**) ER, (**C**,**D**) PR, (**E**,**F**) ER/PR, (**G**,**H**) PAM50 subtype, (**I**) Size, (**J**) Grade, (**K**) Lymph Node Stage, and (**L**) Integrative Cluster. METABRIC cohort was analysed using the one-way analysis of variance (ANOVA) with the post hoc Tukey test. Statistically significant *p*-values shown in **bold**. *** *p* < 0.0001; ** *p* < 0.001; * *p* < 0.01.

Within the histological types, lobular carcinoma (pure or mixed) showed the most *HAO1* CN gain (15/237 6.3%), while *HAO1* CN loss (142/1544, 9.2%) was more common in ductal carcinoma (Table 2; *p* = 0.007). *HAO1* CN and mRNA were not associated with tumour size, grade, or nodal stage (Table 2 and Figure 4I–K).

HAO1 protein was associated with ductal carcinoma (Table 3; *p* = 0.0006). High HAO1 expression was significantly associated with high tumour grade (Table 3; *p* = 0.002), which was primarily due to the association with high mitotic frequency (*p* = 0.0001) and pleomorphism (*p* = 0.001). Whilst there was no association between HAO1 protein and tumour size or nodal stage, high HAO1 expression was correlated with the poor Nottingham Prognostic Index (NPI) group (Table 3; *p* = 0.018).

### 2.4. Bioinformatics Analysis of HAO1 Expression with Invasive Breast Cancer Biological Subtypes

Whilst there was no difference in *HAO1* mRNA expression associated with ER or PR in the METABRIC dataset (Figure 4A,C,E; *p* > 0.05), there was a significant association between high *HAO1 mRNA* expression and ER negative and PR negative tumours in bc-GenExMiner (Figure 4B,D,F; all *p* < 0.0001). There was no association between *HAO1* CN and ER, PR, or HER2 (Table 2). HAO1 protein demonstrated an association with ER (Table 3; *p* = 0.004) but showed no significant association with PR or HER2 status.

Within the PAM50 subtypes, luminal B tumours were more likely to have an *HAO1* gene CN variation but curiously showed either loss or gain of *HAO1* (Table 2; *p* = 0.001). *HAO1* mRNA expression was not associated with the PAM50 subtypes in the METABRIC dataset (Figure 4G; *p* = 0.8), but in the bc-GenExMiner dataset high *HAO1* mRNA expression was observed in basal-like BC (Figure 4H; *p* < 0.0001).

### 2.5. Bioinformatics Analysis of HAO1 in Invasive Breast Cancer and Patient Outcome

**Figure 5:**
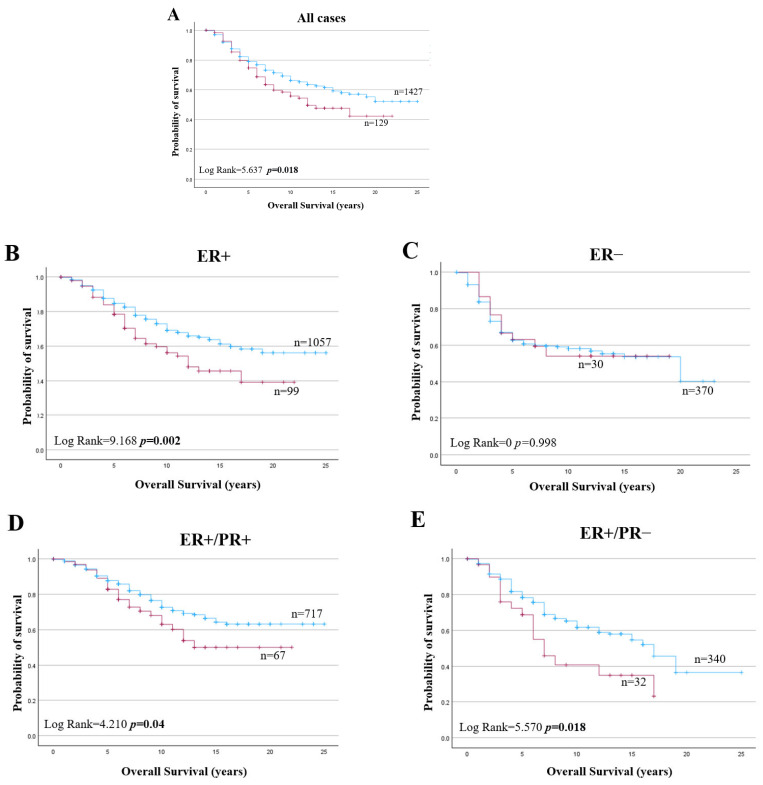
*HAO1* copy number gain (red line), neutral (blue line), and overall survival (OS) in invasive breast cancer (METABRIC): (**A**) all cases, (**B**) ER+, (**C**) ER−, (**D**) ER+/PR+, (**E**) ER+/PR− tumours. Statistically significant *p*-values in **bold**.

**Figure 6:**
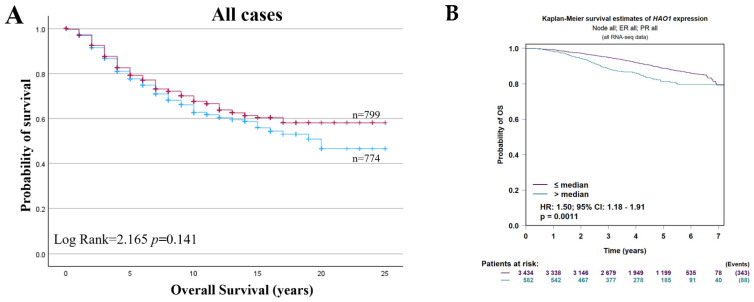

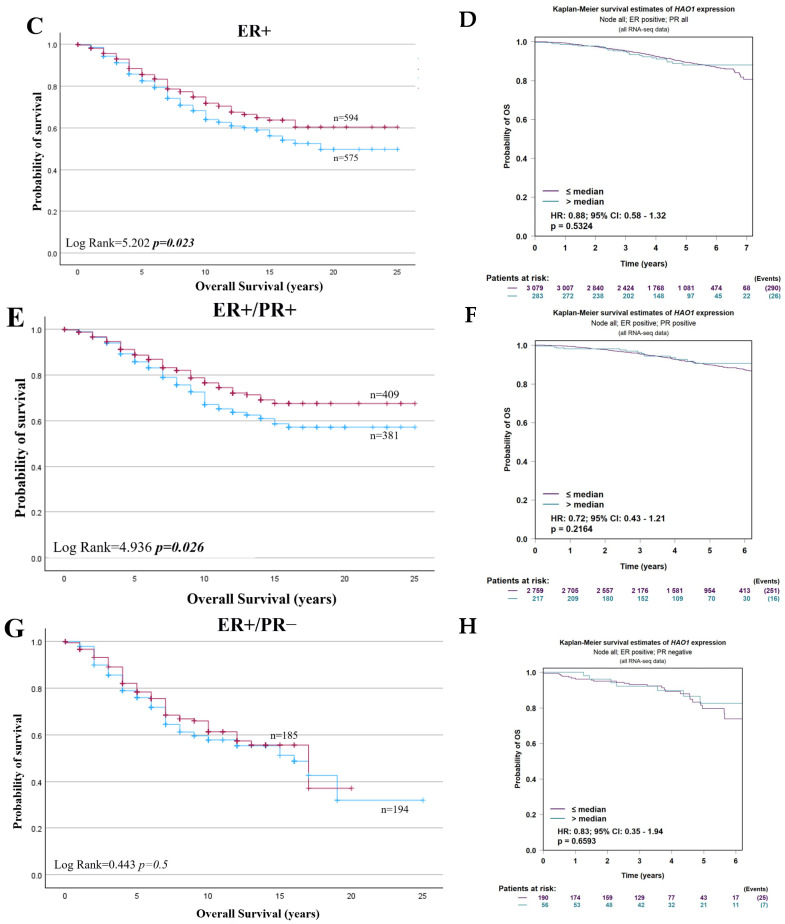
*HAO1* mRNA high (red line), low (blue line), and overall survival (OS) in invasive breast cancer (METABRIC and bc-GenExMiner): (**A**,**B**) all cases, (**C**,**D**) ER+, (**E**,**F**) ER+/PR+, (**G**,**H**) ER+/PR− tumours. Statistically significant *p*-values in **bold**.

**Figure 7:**
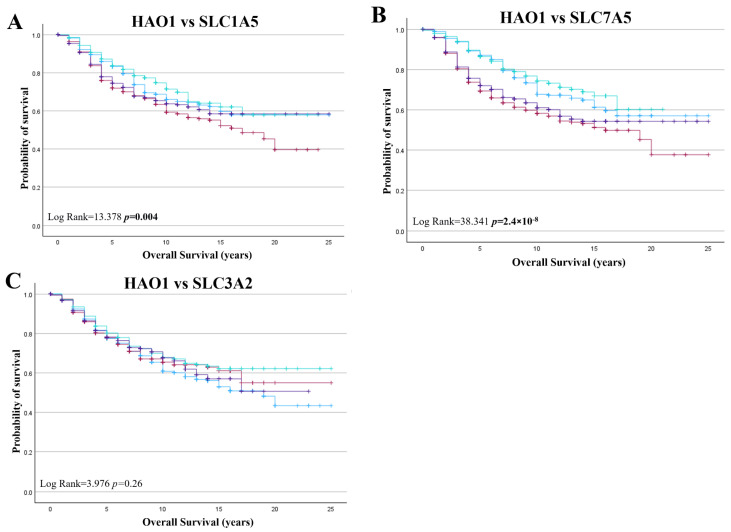
*HAO1* mRNA and patient outcome in invasive breast cancer (METABRIC) (**A**) *SLC1A5*, (**B**) *SLC7A5*, (**C**) *SLC3A2* mRNA expression. low HAO1/low SLC (blue line), low HAO1/high SLC (red line), high HAO1/low SLC (green line), high HAO1/high SLC (purple line). Statistically significant *p*-values shown in **bold**.

*HAO1* CN gain was a poor prognostic marker in all BC cases (Figure 5A; *p* = 0.018). *HAO1* CN gain also predicted poor overall survival for tumours that were ER+, ER+/PR+, and ER+/PR− (Figure 5B,D,E; *p* = 0.002, *p* = 0.040 and *p* = 0.018, respectively) but not in ER negative tumours (Figure 5C; *p* = 0.998). However, *HAO1* CN loss did not show any significant association with overall survival (Supplementary Figure S3). Whilst *HAO1* mRNA showed no association with overall survival in all cases (Figure 6A), high *HAO1* mRNA showed poor survival in ER+ and ER+/PR+ tumours (Figure 6C,E; *p* = 0.023, *p* = 0.026, respectively). High *HAO1 mRNA* combined with high *SLC1A5* and *SLC7A5* mRNA also showed a worse prognosis (Figure 7A,B; *p* = 0.004 and *p* = 2.4 × 10^−8^).

BC patients with *HAO1* hypermethylation at cg19201723 in the initial base pairs after the transcription start site showed a worse patient survival (*p* = 0.044; Supplementary Figure S4B). Furthermore, hypermethylation of *HAO1* in the 3′UTR region, cg19862235, was also associated with poor outcome (*p* = 0.003; Supplementary Figure S4C). The other methylated sites did not predict patient outcome (Supplementary Figure S4A,D).

**Figure 8:**
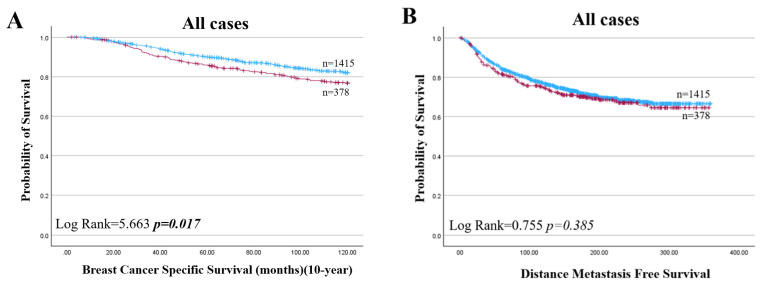

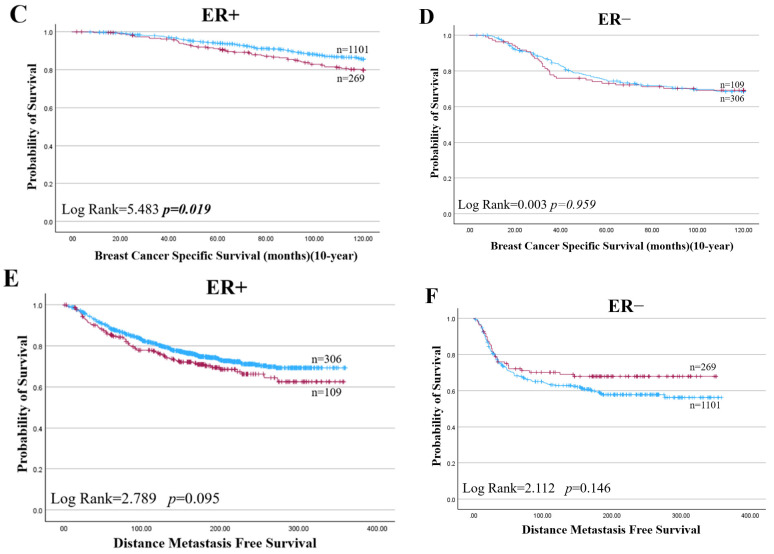
HAO1 protein high (red line), low (blue line), and patient outcome in invasive breast cancer. (**A**,**B**) all cases, (**C**,**E**) ER+, (**D**,**F**) ER− in BCSS and in DMFS. Statistically significant *p*-values shown in **bold**.

High HAO1 protein was also predictive of a shorter BCSS in all cases and specifically for ER+ BC (Figure 8A,C; *p* = 0.017, *p* = 0.019) but not in ER negative cases (Figure 8D; *p* = 0.959). In multivariate analysis, HAO1 protein was a prognostic factor independent of tumour size, nodal stage, and grade in ER+ tumours (Supplementary Table S1).

**Figure 9:**
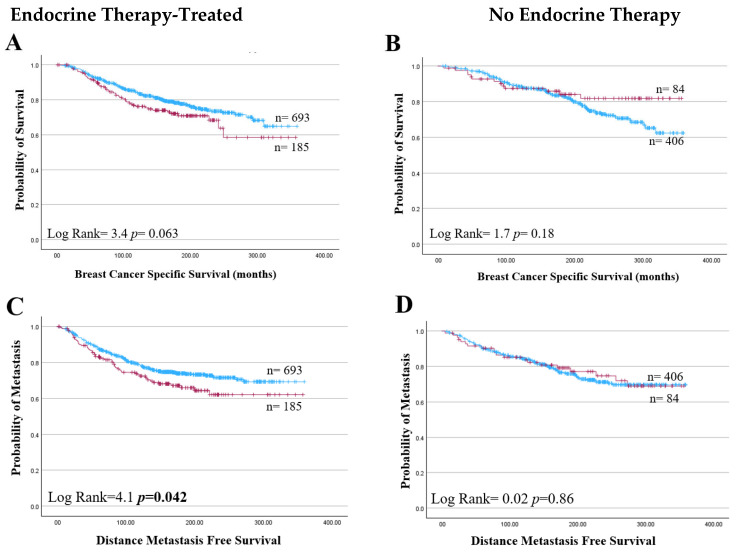
HAO1 protein high (red line), low (blue line), and patient outcome in invasive breast cancer. (**A**,**B**) Breast Cancer Specific Survival, (**C**,**D**) Distance Metastasis-free Survival in ER+ BC patients who received endocrine therapy or without endocrine therapy. Statistically significant *p*-values shown in **bold**.

High HAO1 expression significantly improved DMFS, but not BCSS, in endocrine therapy-treated BC (*p* = 0.042; Figure 9C). On the other hand, the results did not show any significant associations between the HAO1 protein expression levels and survival outcomes in patients, regardless of their treatment with chemotherapy. *HAO1* mRNA expression did not show a significant association with patient outcomes in ER+ or ER- types across tumour size, grade, or stage (Table 4). 

## 3. Discussion

This study explored the prognostic significance of HAO1 expression at various molecular levels—gene CN, mRNA, and protein—and its relationship with clinical outcomes in invasive breast cancer, especially in ER+ subtypes. The findings highlight a potential association between HAO1 and SLCs in luminal BC, suggesting that HAO1 could be a novel prognostic marker and a potential therapeutic target.

BC is a heterogeneous disease, with the distinct molecular subtypes having differences in tumour biology and outcomes for patients [11]. Understanding the metabolic profiles and biological characteristics of these subtypes can aid in identifying potential therapeutic targets and prognostic markers. BC subtypes show different metabolic signatures between luminal and non-luminal BCs. Reprogrammed metabolic enzymes are a hallmark of cancer that can trigger the uncontrolled proliferation of cancer cells. This study provides evidence at both the mRNA and protein levels that HAO1 is a promising metabolic biomarker influencing patient outcomes in luminal BC.

Although endocrine therapy is one of the most effective and well tolerated treatments, identifying biomarkers that predict the effectiveness of endocrine therapy could lead to more effective management treatments for luminal BC patients [12,13]. Therefore, the discovery of additional biomarkers, including SLCs, is essential for optimising the stratification of patients with luminal BC and for guiding more precise and effective targeted therapeutic approaches. Understanding how SLCs contribute to the metabolic reprogramming in luminal BC, particularly in conjunction with HAO1, could provide valuable insights into patient-specific treatment approaches and improve clinical outcomes. In this study, we show that patients with ER+ tumours and a higher expression of HAO1 have predicted shorter DMFS.

Cancer cells are known to undergo metabolic reprogramming to sustain rapid growth and survival under adverse conditions [14]. This includes enhanced glycolysis, increased glutamine metabolism, and alterations in lipid and nucleotide biosynthesis. Glutamine plays a critical role in cancer metabolism, serving as a source of carbon and nitrogen for synthesizing nucleotides, amino acids, and other metabolites [14,15]. It also fuels the tricarboxylic acid (TCA) cycle, contributing to energy production and biosynthetic precursor generation. Although in vivo evidence suggests that HAO1 regulates the TCA cycle, its specific role in glutamine metabolism, particularly with SLCs, and its importance across different BC subtypes remain unclear [16].

HAO1 is crucial in oxalate synthesis, catalysing the oxidation of glycolate to glyoxylate and subsequently to oxalate [16,17,18]. The accumulation of oxalate, driven by HAO1, has been shown to activate the MAPK signalling pathway, a key regulator of cell proliferation, survival, and metastasis. This activation promotes the proliferation of metastatic cancer cells, highlighting HAO1’s role in facilitating tumour growth in lung cancer [16,19].

The relationship between HAO1 and SLCs seems to be particularly important in the context of glutamine metabolism. SLC1A5, SLC7A5, and SLC3A2, key solute carriers, are overexpressed in luminal BC and have been linked to poor patient outcomes. These SLCs enable cancer cells to transport glutamine, fuelling the metabolic processes vital for tumour growth and survival. HAO1, through its role in oxalate synthesis, may interact with this glutamine-driven metabolic reprogramming. This reprogramming involves enhanced glycolysis (the Warburg effect), increased glutamine metabolism, and alterations in lipid and nucleotide biosynthesis [4].

The accumulation of oxalate via HAO1 could alter the redox state and overall metabolic environment of cancer cells, potentially modulating SLC activity or being regulated by the metabolic changes they induce.

The observed correlation between high HAO1 protein levels and poor outcomes, alongside the association of high HAO1 mRNA levels with better survival, suggests that there are complex regulatory mechanisms at play. It is conceivable that the metabolic stress induced by heightened glutamine uptake via SLCs might upregulate HAO1 as a compensatory response. Conversely, HAO1 activity might influence glutamine availability or the cellular redox state, affecting SLC expression or activity. Yet post-translational regulation mechanisms can still be at play in protein expression regarding the complex interactions of the gene within the tumour microenvironment, which the gene and its mRNA will not directly reflect. This may also cause the post-transcriptional regulation that contributes to the differences between mRNA and protein levels [20]. Another possible reason is the tumour microenvironment, so the protein, like any other cellular component, interacts with the surrounding tumour microenvironment, and interactions in the tumour microenvironment may also result in differences in the expression of mRNA and protein [21,22]. The previous study, that builds on providing data that altered glutamine pathways in breast cancer influence the development of specific immune cell subtypes within the tumour microenvironment, showed that reprogramming glutamine metabolism impacts the structure of immune cells [23].

SLCs, through their impact on intracellular nutrient levels and signalling pathways like mTOR, might regulate these mechanisms, affecting how HAO1 is expressed or functions in different pathways.

Our findings also indicate that HAO1 protein levels are not solely determined by the gene CN or mRNA expression. Although a gain in *HAO1* CN was observed in some tumours, it did not correlate with higher *HAO1* mRNA expression, suggesting that other regulatory mechanisms are involved. For example, DNA hypomethylation tends to become more significant as tumours progress or as the malignancy level increases [24,25]. The results demonstrated a negative correlation between *HAO1* mRNA levels and CN, protein, and promoter methylation levels. The differing results obtained in the *HAO1* CN gain, mRNA, and protein highlight how intricate the metabolic signature is. Gene amplification and overexpression of mRNA are, for example, generally linked to more aggressive behaviours in cancer, as shown by different studies [26,27]. Another critical factor in regulating HAO1 protein levels could be protein stability. Knockdown experiments with *SLC1A5*, *SLC7A5*, and *SLC3A2*, which resulted in significant reductions in HAO1 protein levels, suggest that these SLCs may affect HAO1 or influence its degradation. The strong correlation between HAO1 protein expression and these SLCs, particularly SLC7A5, shows a possible regulatory feedback loop where SLCs not only impact glutamine metabolism but also modulate HAO1 protein stability and function. As part of further studies, investigating the effect of a combined knockdown of multiple SLCs and the loss of HAO1 on SLC expression provides a valuable opportunity to better understand the regulatory dynamics between HAO1 and SLCs, providing a clearer understanding of whether loss of HAO1 reciprocally affects SLC expression. Additionally, to strengthen the specificity of the observed relationship between glutamine metabolism, SLCs, and HAO1, future studies may include controls using other solute carriers, such as neutral or cationic carriers.

In summary, this study underscores the importance of HAO1 as a potential biomarker in ER+ BC, particularly in the context of metabolic reprogramming and its interaction with SLCs. The identification of HAO1’s role in the metabolic landscape of luminal BC suggests that targeting the HAO1-SLC axis could offer new therapeutic opportunities. However, further research is needed to fully elucidate the regulatory mechanisms governing HAO1 expression and its impact on BC prognosis. Understanding these interactions will be crucial in developing targeted therapies that exploit metabolic vulnerabilities in luminal breast cancer.

## 4. Materials and Methods

### 4.1. Gene Selection

Differential gene expression analysis based on the negative binomial distribution (DESeq2) was carried out using the TCGA RNA-sequencing BC dataset (The Cancer Genome Atlas Network, 2012). Patients were stratified into quartiles (Q1–4) based on their combined gene expression of *SLC1A5*, *SLC7A5,* and *SLC3A2*. Tumours that showed the highest SLC expression (Q4) were compared to tumours showing the lowest expression (Q1) to identify significantly different gene expression between the two groups (fold change ±2 and FDR-corrected *p*-value < 0.05). These genes were ranked according to those showing the greatest difference in expression. The gene identified as being most strongly associated with high SLCs and, consequently, further explored in this analysis was hydroxyacid oxidase 1 (*HAO1*).

### 4.2. Gene Copy and Expression Analysis

The Molecular Taxonomy of Breast Cancer International Consortium (METABRIC) cohort (n = 1980) utilising cDNA microarrays was assessed for *HAO1* gene copy number (CN) and mRNA expression [28]. *HAO1* mRNA expression was dichotomised into a high and low expression group using the median cut-off log2 intensity value of 5.397, and subsequent associations between the expression groups and various clinicopathological parameters, molecular BC subtypes, and patient outcomes were evaluated. Breast Cancer Gene-Expression Miner (version 5.0) was used to validate *HAO1* gene expression using RNA sequencing datasets (n = 4421), available at (https://bcgenex.ico.unicancer.fr/, accessed 20 May 2024). This statistical tool, which incorporates the SCAN-B and TCGA studies, allowed the evaluation, correlation, differential expression, and prognostic significance of *HAO1* in BC. The dichotomisation of gene expression for prognostic analysis utilised the ‘median’ criterion.

### 4.3. Expression and Methylation Patterns of HAO1 in Breast Invasive Carcinoma

UALCAN (http://ualcan.path.uab.edu, accessed on 20 May 2024) is an extensive online resource designed for the detailed analysis of TCGA data. *HAO1* expression data were retrieved from the TCGA Analysis module of UALCAN using the breast cancer dataset [29]. The MethSurv tool (https://biit.cs.ut.ee/methsurv, accessed on 20 May 2024) was utilized to investigate the DNA methylation of *HAO1* in the TCGA dataset in relation to patient survival or the prognosis of BC patients. DNA methylation levels were dichotomised using the ‘best’ option provided by the tool to optimise the separation of survival outcomes.

### 4.4. Protein Analysis

The expression of hydroxyacid oxidase 1 (HAO1) protein was evaluated in a cohort of 749 early-stage primary operable invasive BC cases treated at Nottingham University Hospitals NHS Trust, Nottingham, UK, between 1989 and 2006. The study was conducted using immunohistochemistry (IHC) and tissue microarrays (TMAs) using pseudo-anonymised samples. Additionally, ten full-face BC tissue sections from the cohort were stained to assess heterogeneity. Clinicopathological profiles of the cohort, including tumour size, grade, nodal stage, vascular invasion status, and molecular subtypes were documented, together with biological profiles and outcome data. Outcome data included survival status, survival time, cause of death, and development, recurrence, and distant metastasis (DM). Definitions of BC-specific survival (BCSS) and distant metastasis-free survival (DMFS) were provided. Treatments given were chemotherapy with the cyclophosphamide methotrexate 5-flurouracil (CMF) regimen and endocrine therapy. Protein expression in ER, PR, HER2, and SLCs with high affinity for glutamine: SLC1A5, SLC7A5, and SLC3A2, were previously determined [30,31].

### 4.5. Western Blotting

The polyclonal antibody specificity of HAO1 (HPA049552, Sigma-Aldrich, Gillingham, UK) was determined using Western blotting in human ER+ (MCF-7, ZR-75-1) and triple-negative (TN) (MDA-MB-436, and MDA-MB-468) BC lysates (American Type Culture Collection; Rockville, MD, USA) at a 1.1000 dilution. The expression of HAO1 was confirmed by the visualisation of three distinct bands at the predicted molecular weights of approximately 20 kDa, 40 kDa, and 60 kDa (Supplementary Figure S5A). To ensure the specificity of the HAO1 antibody, the PrEST Antigen HAO1 (APREST85346, Sigma-Aldrich, UK) was used as per the manufacturer’s recommendations. The mouse anti-β-actin antibody (A5441, Sigma-Aldrich, UK) was utilised at a dilution of 1:5000 to serve as a housekeeping protein, revealing a band at approximately 42 kDa (Supplementary Figure S5A).

### 4.6. siRNA Knockdown of Solute Carriers

To investigate the effects of silencing selected SLCs on HAO1 expression, siRNA transfection was conducted to specifically target the *SLC1A5*, *SLC7A5*, or *SLC3A2* genes, and a scrambled siRNA was used as a negative control (ThermoFisher Scientific, Loughborough, UK, s12918, s15653, s12943, 4390843).

MCF-7 and ZR-75-1 cells were transfected with siRNA using lipofectamine RNAiMAX (Invitrogen, Paisley, UK, 13778150) according to the manufacturer’s protocol. A total of 48 h post transfection, the transfected cells were collected. To evaluate the impact of SLC knockdown, western blotting for SLC1A5, SLC7A5, SLC3A2, and HAO1 was performed on the lysates collected from the transfected cells. The relative expression levels of HAO1 were quantified using Image Studio Lite Ver 5.2 software. The resulting data were analysed using GraphPad Prism 9.4.0. The siRNA efficiencies of SLCs in luminal breast cancer cell lines are shown in Supplementary Figure S6.

### 4.7. Immunohistochemistry

HAO1 protein expression was analysed using IHC on 4 μm TMA sections, employing the Novolink polymer detection system (RE7150-K, Leica Biosystems, Milton Keynes, UK). Heat-induced antigen epitope retrieval was conducted using citrate buffer (pH 6.0) for 20 min in a water bath. The TMAs were incubated with HAO1 antibody at a 1:100 dilution in antibody diluent (RE AR9352, Leica Biosystems, Newcastle upon Tyne, UK) at room temperature for 60 min. Positive control tissue (liver tissue) and negative control (endometrium tissue) were included as specified in the manufacturer’s datasheet (Supplementary Figure S5B,C). Stained TMA sections were scanned using high-resolution digital images with a 3DHistech Pannoramic 250 Flash II at ×20 magnification. Slideviewer software version 2.7 (3DHISTECH Ltd., Budapest, Hungary) was used to view the images.

### 4.8. Immunohistochemical Scoring

The evaluation of HAO1 staining was performed using a semiquantitative method that assessed high resolution digital images of TMA cores. This involved employing a modified histochemical score (H-score) to measure both the intensity of the staining and the percentage of invasive tumours cells that were stained. The staining intensity was evaluated using the following scale: 0 for negative, 1 for weak, 2 for medium, and 3 for strong. The percentage of positively stained tumour cells was estimated subjectively. A pathologist performed blind double scoring independently (AGL), to evaluate concordance. The Interclass Correlation Coefficient (ICC) analysis demonstrated good reliability between the observers (BE and AGL) (0.856). The final score was calculated by multiplying the intensity (0–3), and the percentage of staining (0–100), producing a total range of 0–300.

HAO1 protein expression was dichotomised into high and low expression groups calculating the best cut-off point (>65 H-score) based on the prediction of BCSS using X-tile (Bioinformatics Software version 3.6.1, Yale University, New Haven, CT, USA) [32].

### 4.9. Statistical Analysis

Statistical analysis was performed using Statistical Package for the Social Sciences (SPSS) software Version 29.0.1.0 (SPSS Inc., Chicago, IL, USA). The Chi-square test was performed for determining interrelationships between HAO1 protein expression with clinicopathological parameters and other biological markers. Differences in the mean among multiple continuous variables were assessed using one-way analysis of variance (ANOVA) with a post hoc Tukey multiple comparison test. Survival curves related to patient outcome were analysed using Kaplan–Meier and log-rank tests. Cox regression analysis was used to identify independent prognostic factors. A *p*-value ˂ 0.05 was considered significant.

## Figures and Tables

**Table 1:** Association of HAO1 protein and SLCs in the Nottingham cohort.

	HAO1 Protein	
	Correlation	*p*-Value
**SLC1A5**	0.07	**0.04**
**SLC7A5**	0.13	**0.0001**
**SLC3A2**	0.05	0.18

**Table 2:** The association of *HAO1* gene copy number with clinicopathological and biological markers in invasive breast cancer (METABRIC). Statistically significant *p*-values shown in **bold**.

		*HAO1* Copy Number				
		Loss n (%)	Neutral n (%)	Gain n (%)	χ2 (*p*-Value)	Total n
**Histological Type**						
Ductal carcinoma, No Special Type		24 (2)	1378 (89)	142 (9)	24.4 **(0.007)**	1544
Lobular mixed		0 (0)	81 (90)	9 (10)		90
Tubular		0 (0)	67 (100)	0 (0)		67
Mucinous		2 (4)	44 (96)	0 (0)		46
Medullary		0 (0)	32 (100)	0 (0)		32
Lobular		2 (1)	139 (95)	6 (4)		147
**Tumour Size**						
<2.0 cm		0 (0)	12 (80)	3 (20)	2.8 (0.250)	15
≥2.0 cm		27 (2)	1756 (90)	162 (8)		1960
**Grade**						
1		3 (2)	159 (93)	8 (5)	6.2 (0.188)	170
2		8 (1)	703 (91)	59 (8)		770
3		16 (2)	847 (89)	89 (9)		952
**Lymph Node Stage**						
1		14 (1)	943 (91)	78 (8)	1.8 (0.773)	1035
2		9 (1)	555 (89)	58 (10)		622
3		5 (1)	284 (90)	27 (9)		316
**Receptor Status**						
ER	+	23 (1)	1355 (90)	128 (9)	0.8 (0.660)	1506
	−	5 (1)	432 (91)	37 (8)		474
PR	+	13 (1)	941 (91)	86 (8)	0.4 (0.802)	1040
	−	15 (2)	846 (90)	79 (8)		940
HER2	+	4 (1)	229 (93)	14 (6)	2.7 (0.262)	247
	−	24 (1)	1558 (90)	151 (9)		1733
**PAM50 Subtypes**						
Luminal A		6 (1)	665 (93)	47 (6)	25.6 **(0.001)**	718
Luminal B		14 (3)	417 (85)	57 (12)		488
HER2-E		3 (1)	216 (90)	21 (9)		240
Basal-like		4 (1)	295 (90)	30 (9)		329
Normal-like		1 (1)	189 (95)	9 (5)		199

**Table 3:** Clinicopathological association of HAO1 protein in breast cancer. Statistically significant *p*-values shown in **bold**.

		HAO1 Protein		
		Low n (%)	High n (%)	χ2 (*p*-Value)
**Histological type**				
Ductal carcinoma, No Special Type (NST)		870 (77)	261 (23)	
Lobular including Lobular Mixed		163 (91)	16 (9)	19.5
Metaplastic carcinoma		6 (86)	1 (14)	**(0.0006)**
Other special tumour types		75 (82)	16 (18)	
Mixed NST and other special types		301 (79)	84 (22)	
**Tumour size**				
<2.0 cm		799 (80)	198 (20)	2
≥2.0 cm		616 (77)	180 (23)	(0.156)
**Grade**				
1		226 (81)	54 (19)	12.1
2		569 (83)	121 (17)	**(0.002)**
3		620 (75)	203 (25)	
**Lymph Node Stage**				
1		888 (80)	228 (20)	0.8
2		407 (79)	110 (21)	0.376
3		119 (75)	40 (25)	
**Receptor Status**				
ER	+	1101 (80)	269 (20)	8.4
	−	306 (74)	109 (26)	**(0.004)**
PR	+	814 (79)	213 (21)	0.3
	−	567 (78)	158 (22)	(0.6)
HER2	+	188 (83)	40 (17)	2.1
	−	1191 (78)	333 (22)	(0.138)
**Mitosis**				
1		643 (83)	131 (17)	17.2
2		283 (79)	76 (21)	**(0.0001)**
3		489 (74)	171 (26)	
**Pleomorphism**				
1		39 (87)	6 (13)	
2		520 (83)	106 (17)	12.7
3		856 (76)	266 (24)	**(0.001)**
**Tubule formation**				
1		103 (82)	23 (18)	2.3
2		411 (77)	124 (23)	(0.312)
3		901 (80)	231 (20)	
**Nottingham Prognostic Index**				
Good		484 (82)	107 (18)	8
Moderate		724 (79)	197 (21)	**(0.018)**
Poor		206 (74)	74 (26)	

**Table 4:** *HAO1* mRNA expression and patient outcome in ER+ and ER− breast cancer using multivariate analysis (METABRIC).

*HAO1* mRNA						
	All Cases		ER+		ER−	
	Hazard Ratio (95% CI)	*p*-Value	Hazard Ratio (95% CI)	*p*-Value	Hazard Ratio (95% CI)	*p*-Value
**Tumour Size**	0.839 (0.498–1.411)	0.14	0.613 (0.323–1.163)	0.14	1.676 (0.712–3.947)	0.53
**Grade**	0.822 (0.492–1.373)	0.48	0.598 (0.317–1.127)	0.59	1.661 (0.696–3.964)	0.86
**Nodal Stage**	0.808 (0.486–1.343)	0.33	0.575 (0.308–1.072)	0.32	1.738 (0.722–4.185)	0.68

## Data Availability

Data is contained within the article or Supplementary Material.

## Supplementary Materials

The following supporting information can be downloaded at: https://www.mdpi.com/article/10.3390/ijms252111572/s1.
