# Supplementary material for: Hydroxyacid Oxidase 1, a Glutamine Metabolism-Associated Protein, Predicts Poor Patient Outcome in Luminal Breast Cancer
Source: Int J Mol Sci. 2024 Oct 28;25(21):11572. doi: 10.3390/ijms252111572 (PMC11545845; doi:10.3390/ijms252111572)
Supplement: Supplementary file 1 [file ijms-25-11572-s001.zip › ijms-3252877-supplementary.pdf]

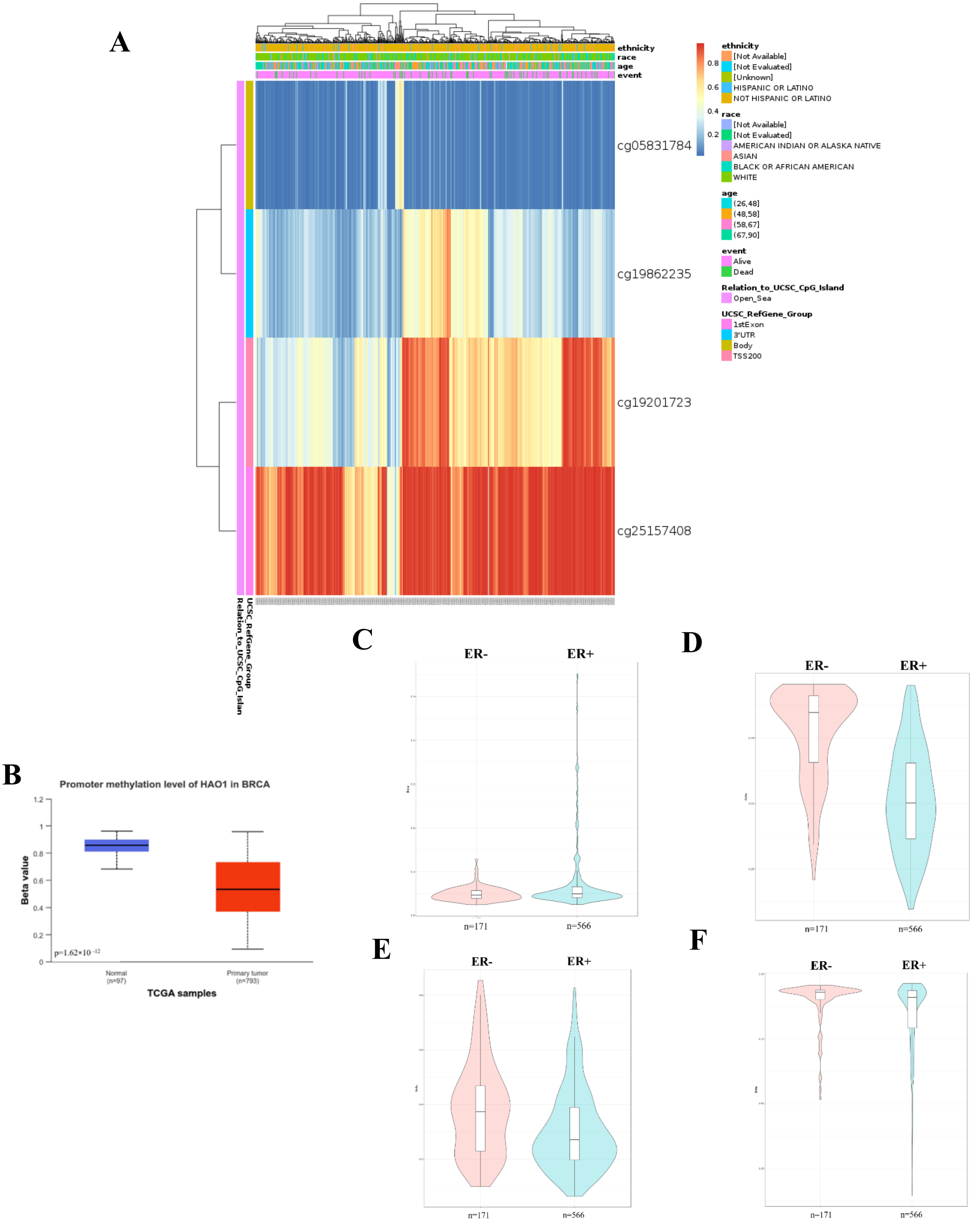

Supplementary Figure S1: *HAO1* methylation in breast cancer: **A)** Heat map of CpG sites, **B)** comparison of levels between breast cancer and normal breast**, C-F)** CpG sites and ER status **C**) cg05831784, **D**) cg19201723, **E**) cg19862235, **F**) cg25157408

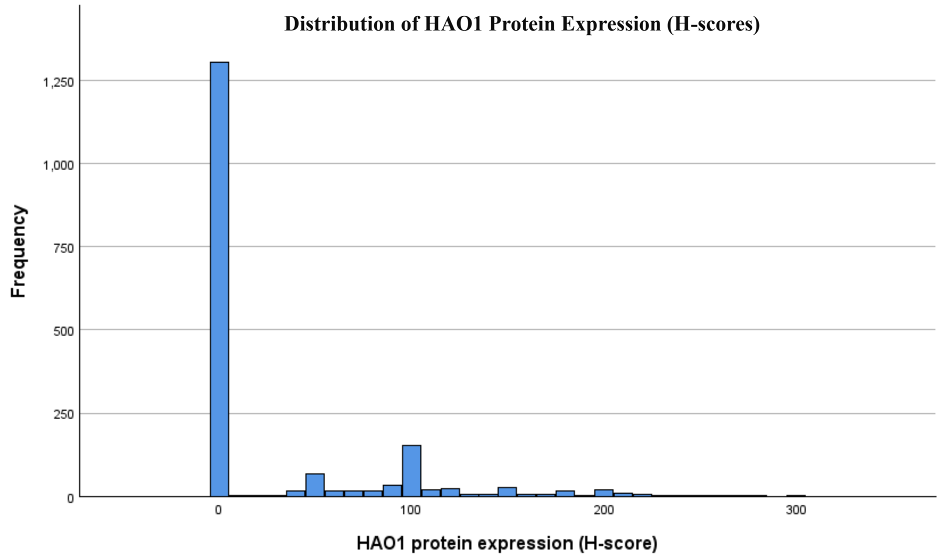

Supplementary Figure S2: Distribution of HAO1 protein expression (H-scores) in breast cancer cases. The X-axis represents the HAO1 protein expression (H-scores) ranging from 0 to 300, while the Y-axis shows the frequency (number of cases)

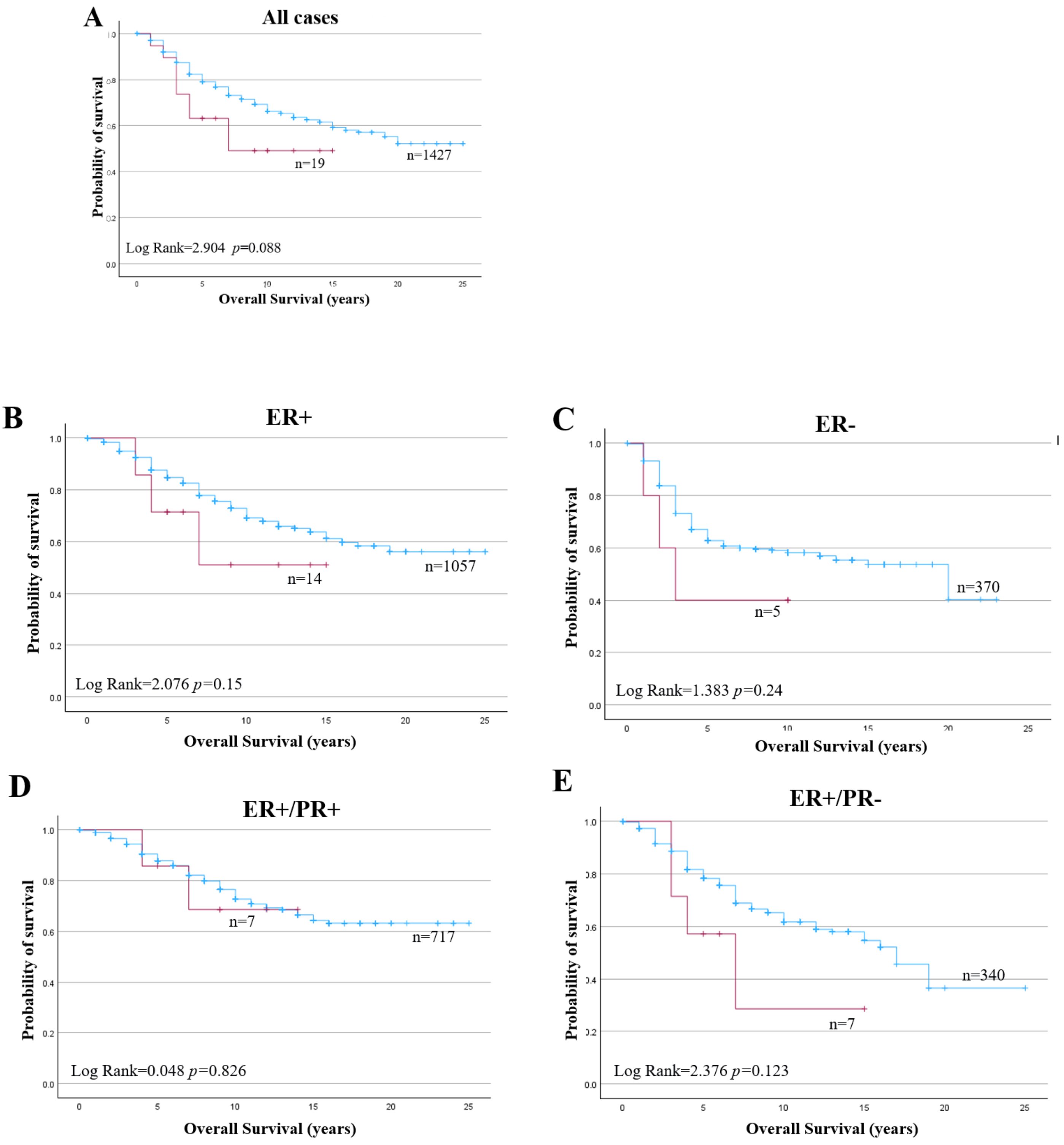

Supplementary Figure S3: *HAO1* copy number loss (red line) and neutral (blue line) in overall survival (OS) in invasive breast cancer (METABRIC): A) all cases B) ER+, C) ER- D) ER+/PR+-, E) ER+/PR-, tumours. Statistically significant *p*-values

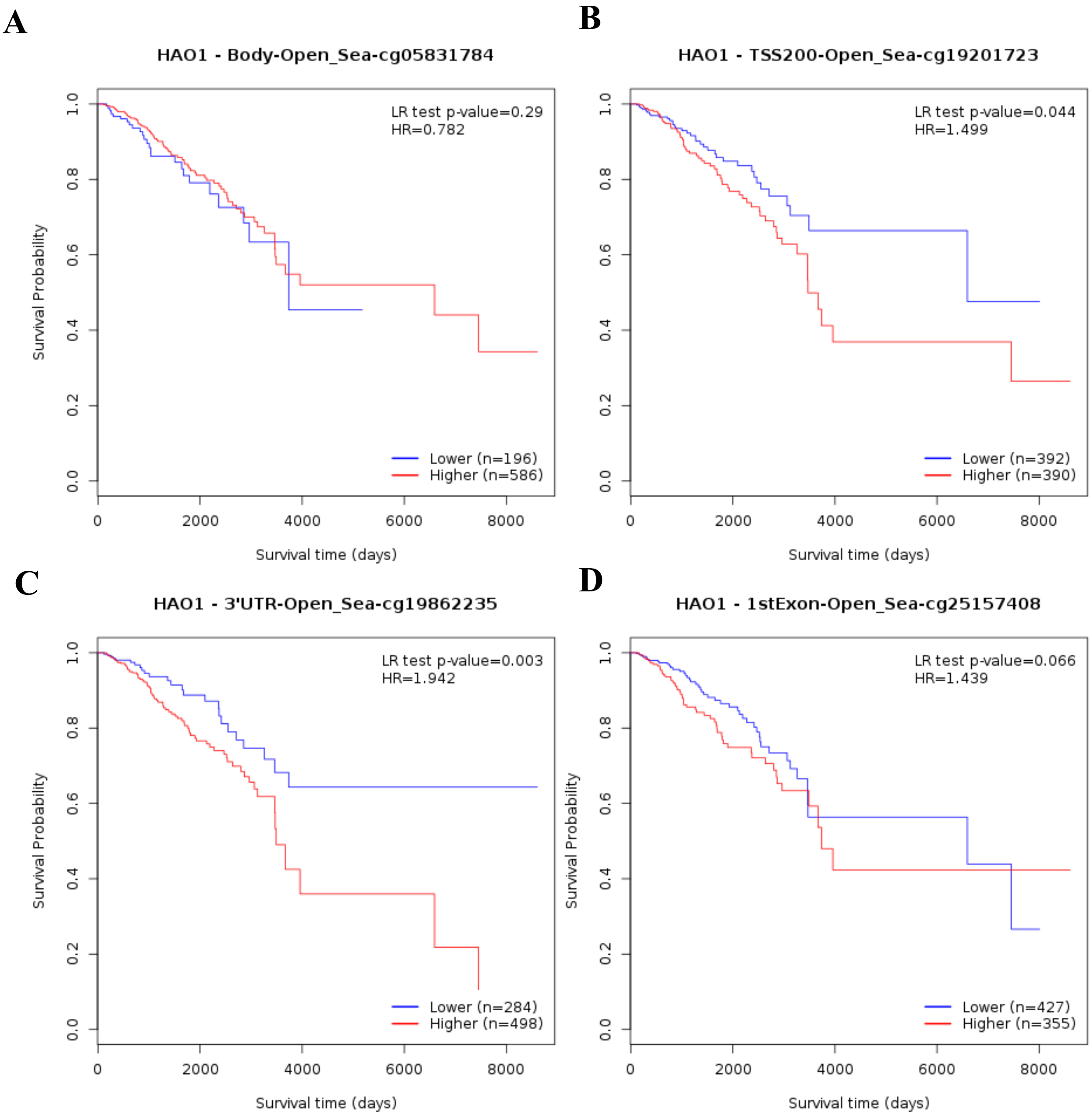

Supplementary Figure S4: *HAO1* methylation in breast cancer and patient outcome: A) cg05831784, B) cg19201723, C) cg19862235, D) cg25157408

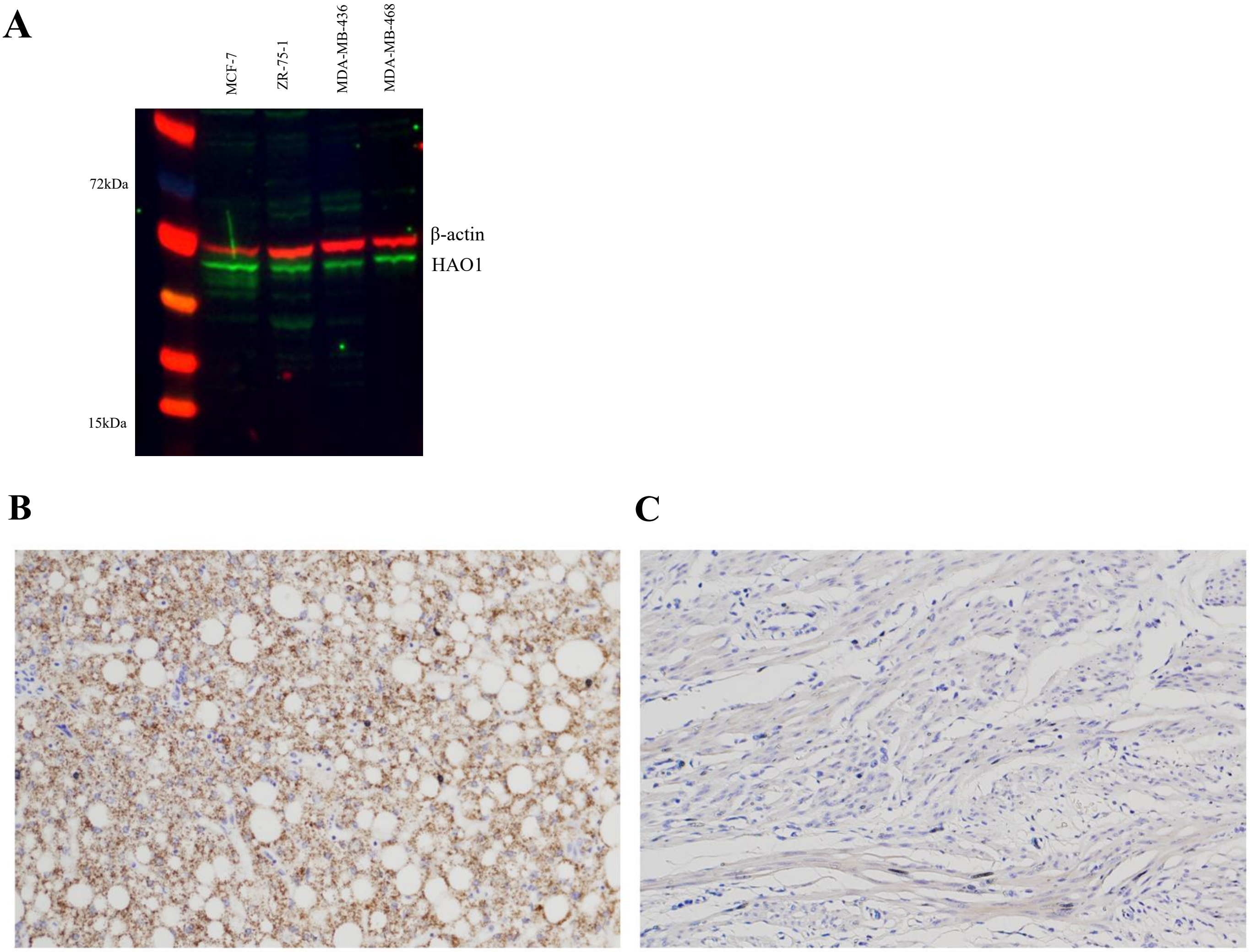

Supplementary Figure S5: A) Western blotting of HAO1 protein expression BC cell lines. HAO1 immunohistochemistry B) positive control (Liver) and C) negative control (endometrium). Magnification 10x

Supplementary Table S1: Multivariate survival analysis of prognostic parameters and HAO1 protein expression in relation to patient outcome using Cox-regression in ER+ vs ER-.

	ER+		ER-	
**Parameters**	**Hazard ratio (95 % CI)**	***p*-value**	**Hazard ratio (95 % CI)**	***p*-value**
**Breast Cancer Specific Survival**				
HAO1	1.034 (0.7-1.3)	0.812	0.883 (0.6-1.3)	0.542
Tumour Size	1.600 (1.2-2)	**<0.001**	1.485 (1.4-2.1)	**0.028**
Grade	1.732 (1.4-2)	**<0.001**	1.010 (0.6-1.4)	0.731
Nodal Stage	1.840 (1.5-2.1)	**<0.001**	1.893 (1.5-2.3)	**<0.001**
**Distant Metastasis Free Survival**				
HAO1	1.159 (0.8-1.4)	0.255	0.768 (0.5-1.1)	0.176
Tumour Size	1.824 (1.4-2.2)	**<0.001**	1.571 (1-2.2)	**0.043**
Grade	1.573 (1.3- 1.8)	**<0.001**	0.916 (0.6-1.3)	0.627
Nodal Stage	1.786 (1.5-2)	**<0.001**	1.813 (1.4-2.2)	**<0.001**

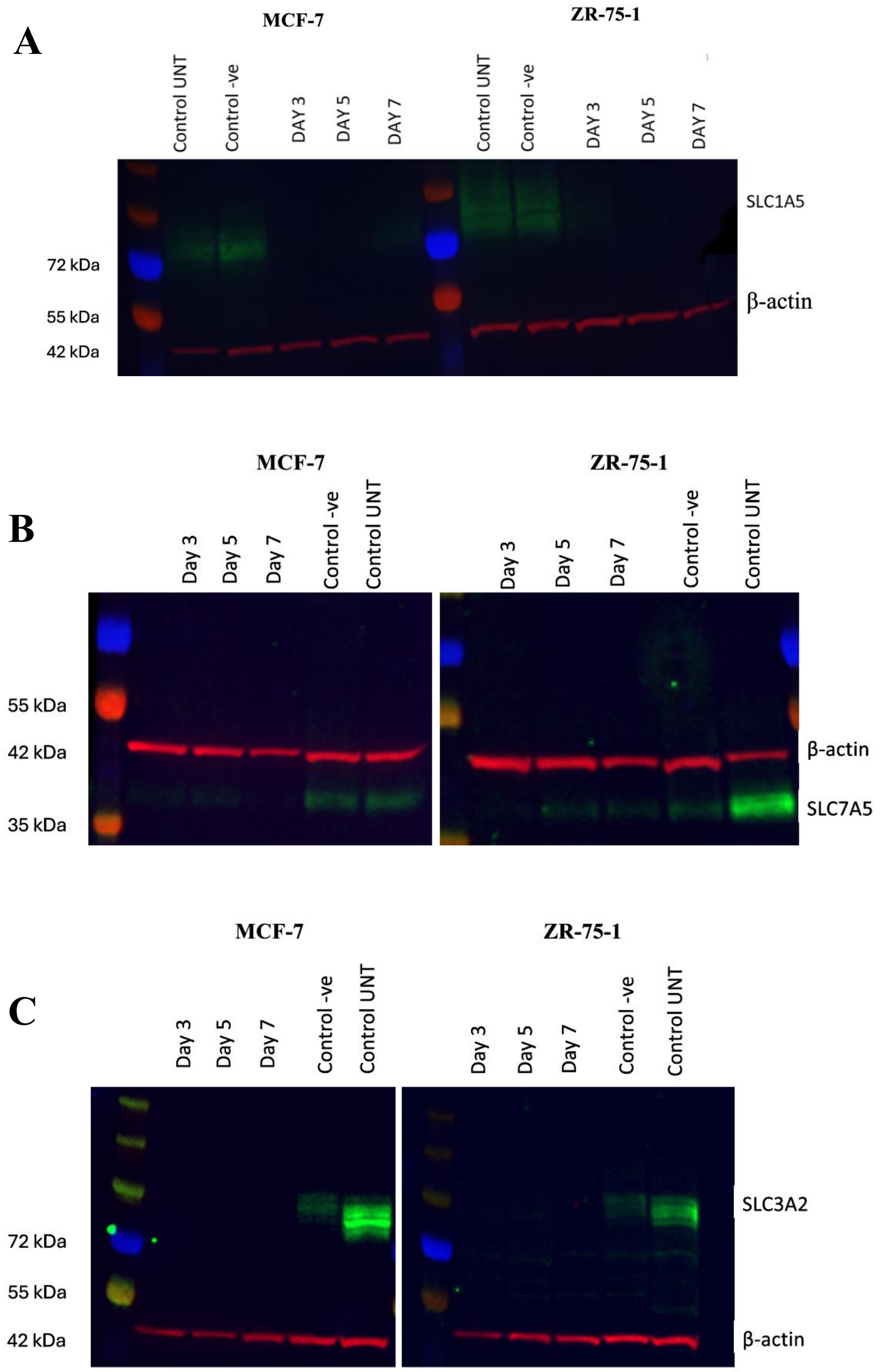

Supplementary Figure S6: siRNA efficiency of SLCs in luminal breast cancer cell lines using siRNA. A) *SLC1A5* B) *SLC7A5* C) *SLC3A2* expression in MCF-7 and ZR-75-1. β-actin was included as a loading control. UNT: Untransfected Control
